# Prevalence and associated factors of early sexual initiation among female youth in East Africa: further analysis of recent demographic and health survey

**DOI:** 10.1186/s12905-022-01895-8

**Published:** 2022-07-22

**Authors:** Samuel Hailegebreal, Girma Gilano, Binyam Tariku Seboka, Habile Sidelil, Shekur Mohammed Awol, Yosef Haile, Atsedu Endale Simegn, Firehiwot Haile

**Affiliations:** 1 Department of Health Informatics, School of Public Health, College of Medicine and Health Sciences, Arba Minch University, Arba Minch, Ethiopia; 2 Department of Health Informatics, School of Public Health, College of Medicine and Health Sciences, Dilla University, Dilla, Ethiopia; 3 Arba Minch College of Health Science, Arba Minch, Ethiopia; 4 Department of Health Informatics, College of Medicine and Health Sciences, Wollo University, Dessie, Ethiopia; 5 College of Medicine and Health Sciences, School of Public Health, Arba Minch University, Arba Minch, Ethiopia; 6 Department of Anesthesia, College of Medicine and Health Sciences, Wachemo University, Hosaena, Ethiopia

**Keywords:** Early sexual initiation, Female youth, East Africa

## Abstract

**Background:**

Early sexual initiation is one of the risky sexual practices. Early sexual beginning is associated with an increased risk of HIV/AIDS, sexually transmitted infections (STIs), unexpected pregnancies, unsafe abortion, premature deliveries, and psychosocial issues. However, there is still a lack of evidence, particularly in East Africa, where community-level factors are not investigated. Therefore, this study aimed to estimate the pooled prevalence and to identify associated factors of early sexual initiation among female youth in Eastern Africa.

**Methods:**

A total weighted sample of 49,716 female youth was included in this analysis. STATA version 14 software was used for data extraction, recoding, and analysis. A multilevel binary logistic regression model was fitted to identify determinants of early sexual initiation in the region. Finally, Adjusted Odds Ratio (AOR) with a 95% Confidence Interval (CI) was reported to declare the factors that are significantly associated with early sexual initiation.

**Result:**

The prevalence of early sexual initiation in East Africa was 21.14% [95% CI: 20.00%, 21.50%]. In the multivariable multilevel analysis; being age 20–24 years [AOR = 0.65: 95% CI; 0.61, 0.69], primary [AOR = 0.73: 95% CI; 0.67, 0.78], secondary &above education [AOR = 0.30: 95% CI; 0.27,0.33], married [AOR = 1.85: 95% CI; 1.73,1.97], middle wealth [AOR = 0.78: 95% CI; 0.72,0.84], richest [AOR = 0.74: 95% CI; 0.68,0.80], and reading newspaper [AOR 0.77: 95% CI;0.71,0.83] were significantly associated with early sexual initiation.

**Conclusion:**

The study revealed that early sexual initiation among female youth was high in East Africa. Educational status, respondent age, marital status, wealth index, age at first cohabitation, contraceptive use, reading newspaper, and place of residence were associated with early sexual initiation. Therefore, the survey findings will help policymakers, as well as governmental and non-governmental organizations, design the most effective interventions. Moreover, strengthening information, education, and wealth status are important intervention areas to delay the age of early sexual debut.

## Background

Early sexual initiation is defined as an experience of first intercourse before 15 years of age [1–3]. According to evidence, youth who engage in early sexual intercourse, are exposed to multiple sexual partners, and use incorrect or irregular condoms are at risk of HIV/AIDS, sexually transmitted infections (STIs), unexpected pregnancies, unsafe abortion, early births, and psychosocial problems. [4–6].Early sexual initiation exposes young people to a variety of sexual and reproductive health issues [7, 8]. Female youth, in particular, are exposed to pregnancy and childbirth difficulties (such as fistula or even death), which are exacerbated by physiological immaturity to engage in sexual intercourse and give birth [9–11].

Early sexual activity is still a persistent issue with harmful psychological and health consequences. The age at sexual debut differs from country to country and individual, and is influenced by a variety of circumstances [12–14]. Early sexual debut among youths is influenced by a variety of factors, including:-Age, residence, religion, educational status, viewing pornographic, alcohol drinking, khat chewing, media content and social/religious ceremonies were the factors associated with early sexual initiation [15–19]. Furthermore, wealth index, media exposure, employment status, comprehensive HIV knowledge, and age at first marriage are all factors that contribute to an early sexual debut [2, 20–22].

According to data from developing-country demographic health surveys, approximately 10% of young girls became mothers at the age of 16, with the majority coming from Sub-Saharan Africa, South Central and South-Eastern Asia [2, 23]. According to studies conducted in various parts of the world, the prevalence of early sexual debut 9.8% in Malaysia [24], 18.1% in China [25], and 58.6% in Caribbean Countries [26]. Besides, early sexual debuts are common in African settings, ranging from 26.8% in Nigeria [27] to 55% in Ghana [28]. Despite studies on the prevalence and associated factors of early sexual initiation have been undertaken in various east African countries [1, 2, 29, 30], there is limited evidence on the region's pooled prevalence and associated factors of early sexual beginning. Although various studies on early sexual initiation have been conducted, the majority of them have failed to take into account community-level factors that may influence the possibility of early sexual initiation.

Therefore, this study aimed to estimate the pooled prevalence and to identify associated factors of early sexual initiation in East Africa based on the pooled nationally representative Demographic and Health Surveys (DHS). As a result, the findings of this study could assist policymakers, as well as governmental and non-governmental organizations, in developing programs and interventions addressing early sexual debut and related consequences.

## Methods

### Data source and participants

The Demographic and Health Survey (DHS) data were pooled from the 12 East African countries from 2008 to 2018 (Burundi, Ethiopia, Comoros, Uganda, Rwanda, Tanzania, Mozambique, Madagascar, Zimbabwe, Kenya, Zambia, and Malawi). Mayotte, Reunion, South Sudan, Djibouti, Seychelles, and Mauritius were removed due to a lack of DHS history. Furthermore, Eritrea and Sudan were not included due to the long period since their last DHS. It was carried out on the basis of a two-stage stratified sampling process. In the first stage, Enumeration Areas (EAs) were assigned at random to their respective clusters. In the second stage, households were selected. The source population were all-female youth (15–24 years old) irrespective of their sexual activity across the east African countries whereas the study populations were all-female youth (15–24 years old) irrespective of their sexual activity in the survey period in the selected Enumeration Areas (EA). The detailed sampling procedure was presented in the full DHS report. A total of 49,716 weighted female youth who have ever experienced sexual intercourse were considered for this analysis.

### Variables of study

In this study, the outcome variable was (early sexual initiation) dichotomized as (Yes/No). Youth who initiated sexual intercourse before the age of 15 years were considered as those experienced an “early sexual initiation [31]. If youth were initiated sexual intercourse before the age of 15 years coded as “1”, otherwise coded as “0”.

Individual and community-level variables were considered as independent variables in this study. Individual-level factors include respondent age, marital status, literacy, contraceptive use, educational status, wealth index, media exposure, employment status, and age at first marriage. The community-level factors were residence and country.

### Data management and statistical analysis

STATA version 14 software was used for data extraction, recoding, and analysis. To restore the data's representativeness and produce a reliable estimate and standard error, the data were weighted. A multilevel logistic regression analysis was employed since the hierarchical structure of the DHS data contradicts the independent assumptions of the ordinary logistic regression model. Whether there was clustering, the Interclass Correlation Coefficient (ICC) and Median Odds Ratio (MOR) were calculated. The null model (without explanatory variables), model I (with individual-level factors), model II (with community-level factors), and model III (with both individual and community-level factors at same time) were used in this analysis. Models were compared using deviance (-2LL), and the best-fitted model was selected as the one with the lowest deviance. In the bi-variable analysis, variables having a p-value of 0.25 were considered for multivariable analysis. Finally, factors having a *P*-value of 0.05 were considered a significant factor associated with early sexual initiation in the multivariable analysis.

## Results

### Socio-demographic characteristics

A total of 49,716 female youth were included in this study. Among this, 38,273 (76.98%) were rural residents, and more than half (80.68%) were between the ages of 20–24 years. The majority of the study participants were from Kenya, Malawi, Burundi, and Uganda. About 57.86% had completed primary school, and 47.56% came from poor families. More than half of the youth (61.24%) were unmarried, and 59.00% did not use any form of contraception (Table 1).

**Table 1:** Socio-demographic characteristics of study participant

Variables	Weighted frequency	Percent
Age		
15–19	9606	19.32
20–24	40,110	80.68
Age at cohabitation		
< 15	11,623	23.38
15–19	31,619	63.60
20–24	6474	13.02
Reading newspaper		
No	40,489	81.44
Yes	9227	18.56
Listing radio		
No	19,891	40.01
Yes	29,825	59.99
Watching TV		
No	35,503	71.41
Yes	14,213	28.59
Place of residence		
Urban	11,443	23.02
Rural	38,273	76.98
Educational level		
No-education	7676	15.44
Primary	28,764	57.86
Secondary and above	13,276	26.70
Marital status		
Single	19,269	38.76
Married	30,447	61.24
Wealth index		
Poor	23,646	47.56
Middle	9668	19.45
Rich	16,402	32.99
Working status		
No	23,875	48.02
Yes	25,841	51.98
Contraceptive use		
No	29,330	59.00
Yes	20,386	41.00
Country		
Burundi	5429	10.92
Ethiopia	2425	4.88
Kenya	11,142	22.41
Comoros	1460	2.94
Madagascar	4091	8.23
Malawi	6280	12.63
Mozambique	3841	7.73
Rwanda	1552	3.12
Tanzania	3114	6.26
Uganda	5092	10.24
Zambia	3375	6.79
Zimbabwe	1916	3.85

### Prevalence of early sexual initiation in East Africa

**Figure. 1:**
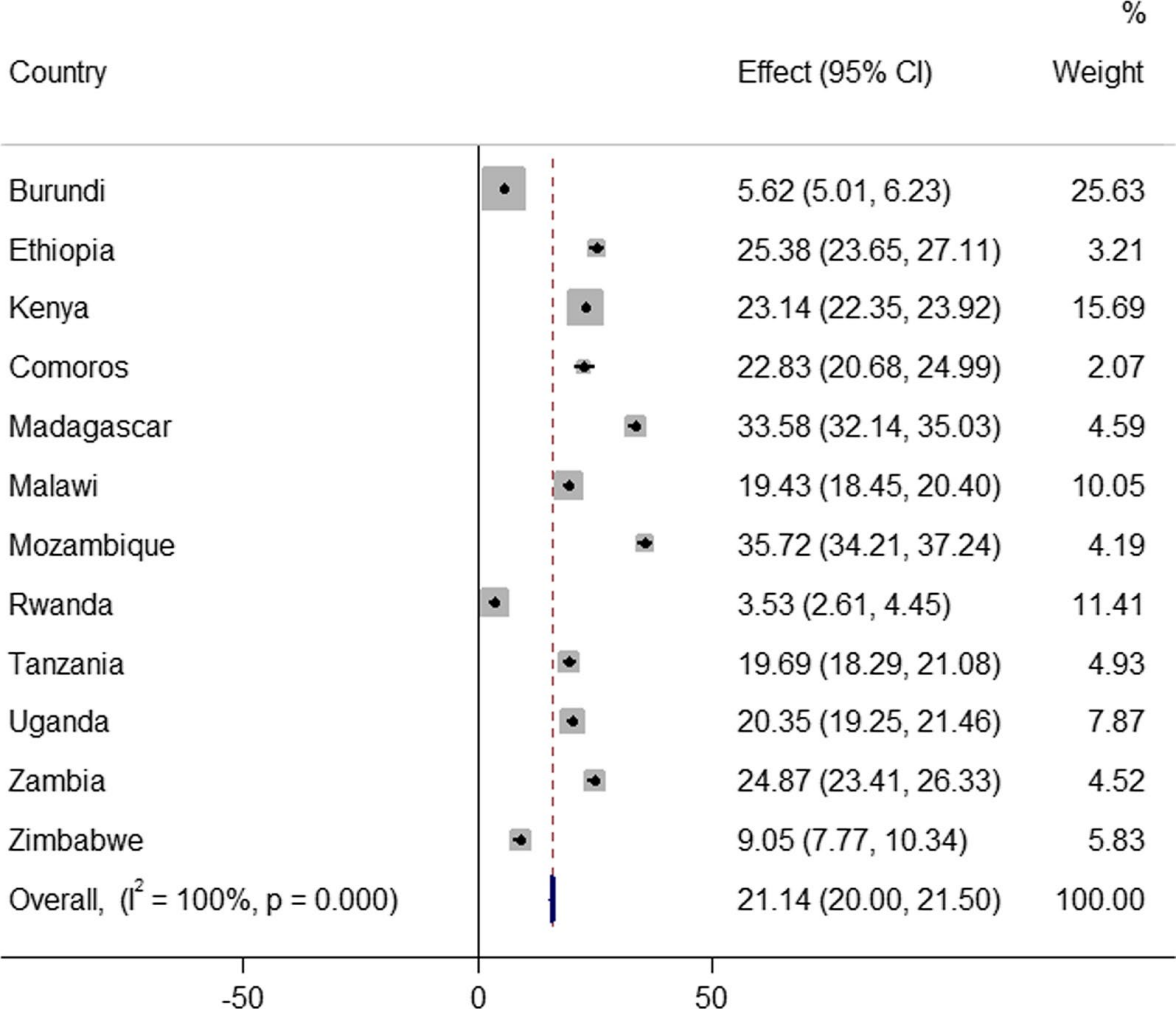
Prevalence of early sexual initiation among youth in eastern Africa countries

The pooled prevalence of early sexual initiation in East Africa was 21.14% [95% CI: 20.00%, 21.50%] with the highest early sexual initiation in Mozambique (35.72%) and the lowest early sexual initiation in Rwanda (3.53%) (Fig. 1).

### Factors associated with early sexual initiation in East Africa

#### Random effect (measures of variations) results

The ICC value in the null model was 0.18, implying that about 18% of the total variation in female youth sexual initiation was due to the clustering effect, while the remaining 82% variation in sexual initiation was due to between-individual variability. Besides that, the MOR value was 2.25 (95% CI: 2.17, 2.36), indicating that early sexual initiation varied significantly between clusters. The final model (Model III) suited the best in terms of model fitness because it had the highest log likelihood and lowest deviance.

#### Multi-level fixed effects (measures of associations) analysis

In the multivariable multilevel binary logistic regression analysis age of respondent, educational status, marital status, wealth status, age at first cohabitation, contraceptive use, reading newspaper, residence, and country were found to be statistically associated with early sexual initiation.

In this study, the odds of early sexual initiation were 35% less likely among youth aged 20–24 years (AOR = 0.65, 95% CI: 0.61, 0.69) than among youth aged 15–19 years. Youth who attained primary and secondary &above education had decreased the odds of early sexual initiation by 27% (AOR = 0.73, 95% CI: 0.67, 0.78) and 70% (AOR = 0.30, 95% CI: 0.27, 0.33) than youth who had no formal education respectively. Married youth had 85% (AOR = 1.85, 95% CI: 1.73, 1.97) higher odds of early sexual initiation as compared with unmarried youth. The odds of early sexual initiation among youth in the middle and richest households were reduced by 22% (AOR = 0.78, 95% CI: 0.72–0.84) and 26% (AOR = 0.74, 95% CI: 0.68–0.80), respectively, compared to youth in the poorest household. When compared to those who did not read the newspaper, youth who read the newspaper were 23% less likely to have early sexual initiation (AOR 0.77, 95% CI 0.71–0.83).Youth who used contraceptives had 1.19 times [AOR = 1.19; 95% CI: 1.28, 1.55] higher odds of early sexual initiation as compared to their counterparts. Youth who began first cohabitation at the ages of less than 15 years and 15–19 years were 13 times (AOR = 13; 95% CI: 11.94 -15) and 1.37 times (AOR = 1.37; 95% CI: 1.23–1.53) more likely to experience early sexual initiation than those who began cohabitation at the ages of 20–24 years, respectively.

Regarding to place of residence youth from urban resident had 16% more likely (AOR = 1.16, 95% CI: 1.07, 1.26) early sexual initiation than rural resident. Furthermore, youth who live in Kenya, Comoros, Madagascar, Malawi, Mozambique, Tanzania, Uganda, and Zambia were 7.6 (AOR = 7.6; 95% CI: 5.66–10.14), 7.5(AOR = 7.5; 95% CI: 5.45–10.44), 9.9 (AOR = 9.9; 95% CI: 7.40–13.29), 6.3 (AOR = 6.4; 95% CI: 4.66–8.37), 17 (AOR = 17; 95% CI: 12.78–22.96), 7.7 (AOR = 7.7; 95% CI: 5.67–10.31), 9.4 (AOR = 9.4; 95% CI: 6.99–12.49), 7.3 (AOR = 9.4; 95% CI: 5.43–9.83), and 7.3 (AOR = 9.4; 95% CI: 5.43–9.83), times higher odds of early sexual initiation than Rwanda respectively (Table 2).

**Table 2:** Multilevel binary logistic regression analysis of factors associated with early sexual initiation in East Africa

Variables	Null-Model	Model-I	Model-II	Model-III
Age				
15–19		1		1
20–24		0.60 [0.56–0.63]**		0.65 [0.61–0.69]**
Age at cohabitation				
< 15		14 [12.75–16.02]**		13.4 [11.9–15.0]**
15–19		1.50 [1.35–1.67]**		1.38 [1.23–1.53]**
20–24		1.50 [1.84–2.08]*		1
Marital status				
Single		1		1
Married		1.96 [1.84–2.08]		1.86 [1.73–1.97]*
Working status				
No		1		1
Yes		0.89 [0.85–0.94]		1.02 [0.95–1.08]
Wealth index				
Poor		1		1
Middle		0.81 [0.75–0.87]		0.79 [0.73–0.85]*
Rich		0.81 [0.74–0.85]		0.76 [0.70–0.82]*
Educational status				
No education		1		1
Primary		0.76 [0.71–0.82]*		0.73 [0.67–0.78]**
Secondary and above		0.31 [0.28–0.34]*		0.31 [0.27–0.33]**
Reading newspaper				
No		1		1
Yes		0.78 [0.72–0.85] **		0.77 [0.71–0.83]**
Listing radio				
No		1		1
Yes		1.04[0.98–1.10]		0.95[0.89–1.01]
Watching TV				
No		1		1
Yes		1.01 [0.94–1.08]		0.94 [0.87–1.01]
Contraceptive use				
No		1		1
Yes		1.07 [1.01–1.13]*		1.20 [1.12–1.26]**
Place of residence				
Rural			1	1
Urban			0.76 [0.72–0.81]	1.16 [1.07–1.26]**
Country				
Burundi			1.57 [1.17–2.10]**	1.8 [1.34–2.47]**
Ethiopia			7.7 [5.80–10.26]**	4.8 [3.56–6.61]**
Kenya			7.9 [6.01–10.43]**	7.6 [5.66–10.14]**
Comoros			9.8[7.29–13.19]**	7.5 [5.45–10.44]**
Madagascar			13 [10.02–17.40]**	9.9 [7.40–13.29]**
Malawi			6.2 [4.69–8.15]**	6.3 [4.66–8.37]**
Mozambique			15 [11.77–20.45]**	17 [12.78–22.96]**
Rwanda			1	1
Tanzania			6 [4.61–8.13]**	7.7 [5.67–10.31]**
Uganda			6.8 [5.13–8.92]**	9.4 [6.99–12.49]**
Zambia			8 [6.15–10.75]**	7.30 [5.43–9.83]**
Zimbabwe			2.4 [1.76–3.30]**	3.14 [2.24–4.40]**
Random effect				
Community variance	0.72	0.69	0.68	0.66
ICC%	18.05%	17.14%	17.26%	16.89%
MOR	2.20	2.14	2.13	2.09
Model comparison				
Log likelihood	−25,312.34	−20,697.13	−24,127.53	−19,906.35
Deviance	50,624.68	41,394.26	48,255.06	39,812.70

*Key: 1: Reference group; *p*-value 0.05–0.01 *: *p*-value < 0.01 **

## Discussion

This study aimed to assess the pooled prevalence and associated factors of early sexual debut in east Africa using the recent demographic and health survey data. The pooled prevalence of female youth East African countries was 21.14% [95% CI: 20.00%, 21.50%] with the highest prevalence in Mozambique (35.72%) and the lowest prevalence in Rwanda (3.53%). The finding was higher than previous studies conducted in South Africa (8.9%) [32], Peru (17.1%) [33] sub-Saharan Africa [1], Ethiopia (17.9%) [34], Caribbean countries [35]. This could be the period when trends in globalization and new technologies are having a growing impact on young female sexual health. Furthermore, we used a large sample size and included people from a wide range of sociocultural and socioeconomic backgrounds.

In the multilevel multivariable logistic regression analysis age, educational level, marital status, wealth status, age at first cohabitation, contraceptive use, newspaper reading, residence, and living countries were determinants of early sexual initiation in the East Africa Countries.

The odds of early sexual initiation were less likely among female aged 20–24 years than among female aged 15–19 years, which consistent with study in Africa [12, 36–38]. This could be due to cultural behaviors such as early marriage and abduction, which were popular at the time. Furthermore, the disparity between the two age-group cohorts may be attributed to the demise of youth-friendly health services.

As the level of education increases, the likelihood of experiencing early sexual initiation decreases. The result was supported by previous studies [39–41]. The possible explanation is that when youth have a better educational level, they are more aware of the potential problems associated with early sexual intercourse and may prevent themselves from involvement. Besides that, education may result in changes in behaviors that reduce possible risks such as substance use, which may expose them to early sexual initiation.

Being married had higher odds of early sexual initiation than youth who were not in a union. This finding was supported by a studies done in Africa [39, 42, 43]. This could be engaged in early marriage before the age of 15 years, which is a potential situation for female to experience sexual activity at an early age.

Female youth from middle and high-income households were less likely to participate in early sex than youth from lower-income households. This is consistent with the study conducted in Africa [28, 39, 44]. This might be females from low-income families may participate in earlier sexual relations to obtain money and other benefits, whereas rich people have good health-seeking behavior, awareness of lifestyle determinants, and family traits.

Youth who started their first cohabitation at the ages of less than 15 years and 15–19 years were more likely to have early sexual initiation than those who started cohabitation at the ages of 20–24 years, respectively this finding was supported by study in Ghana [38].This study found that youth females who used contraception had a higher risk of early sexual debut than their counterparts, which is consistent with other studies in Africa [2]. This could be because young people who take contraception are less likely to become pregnant and have more confidence to begin sexual activity at a young age.

Young girls who read newspapers or magazines are less likely to engage in sexual intercourse at a young age, which is in line with previous findings [2, 45–47]. This could be because participants who read newspapers are more likely to be aware of the risks of early sexual initiation.

Furthermore, youth from rural areas had a lower likelihood of early sexual initiation compared to urban youth; this finding was consistent with previous studies [48–50]. This could be attributed to urban females being exposed to alcohol, substances, and pornographic materials, which leads to an early sexual debut. Pornographic media can arouse emotional and physiological desire for sex, pushing the girl to put what she has seen to the test, leading to early sexual practice [47, 51].

In East African countries, country residency has a significant impact on early sexual initiation. Rwanda was selected as a reference for its low percentage of early sexual beginning. This could be ascribed to altering conventional norms as a result of globalization, which causes changes in socio-demographic characteristics such as religion, media exposure, education, and youths people's socioeconomic situation [52–54]. This difference could be the reason why young females' sexual debuts differ across the country.

### Strength and limitation of the study

As strength, weighted nationally representative data from 12 eastern African countries with a substantial sample size were used in the study. Besides, the study was based on an advanced (multilevel) model, by taking into account the clustering effect, to get reliable standard error and estimate. However, this study had certain limitations. First, because the data came from a cross-sectional survey, no causal conclusions regarding whether characteristics connected with early sexual initiation could be drawn. Second, because females tend to under-report sexual behavior, data obtained from self-reported sexual behaviors may be skewed or misleading.

## Conclusion

This study showed that early sexual initiation was high among female youth in East Africa. Country, urban residence, age at first cohabitation, married marital status, and contraceptive use was positively associated with early sexual initiation. Whereas, educational status, age group 20–24 years, newspaper reading, middle and rich wealth status was negatively associated with early sexual initiation. Therefore, the survey findings will help policymakers, as well as governmental and non-governmental organizations, design the most effective interventions. Moreover, strengthening information, education, and wealth status are important intervention areas to delay the age of early sexual debut. Besides, African countries should develop an integrated strategy for improving preventing early sexual initiation among youth with low socioeconomic level.

## Data Availability

Data we used for this study are publicly available in the MEASURE DHS program and you can access it from www.measuredhs.com after explaining the objectives of the study. Then after receiving the authorization letter, the data is accessible and freely downloaded.

## Abbreviations

AOR: Adjusted odds ratio
CI: Confidence interval
DHS: Demographic and health survey
ICC: Intra class correlation coefficient
LLR: Log-likelihood ratio
MOR: Median odds ratio
OR: Odds ratio
PVC: Proportional change in variance
WHO: World Health Organization
